# Using New Technologies to Analyze Gut Microbiota and Predict Cancer Risk

**DOI:** 10.3390/cells13231987

**Published:** 2024-12-01

**Authors:** Mohammad Amin Hemmati, Marzieh Monemi, Shima Asli, Sina Mohammadi, Behina Foroozanmehr, Dariush Haghmorad, Valentyn Oksenych, Majid Eslami

**Affiliations:** 1 Student Research Committee, Semnan University of Medical Sciences, Semnan 35147-99442, Iran; hemmati.m.amin@gmail.com (M.A.H.); behinaforouzanmehr1377@gmail.com (B.F.); 2 Department of Basic Science, Faculty of Pharmacy and Pharmaceutical Science, Tehran Medical Science, Islamic Azad University, Tehran 19395-1495, Iran; mmomeni1976@gmail.com; 3 Faculty of Medicine, Semnan University of Medical Sciences, Semnan 35147-99442, Iran; shimaasl1378@gmail.com (S.A.); sinamohammadi911@yahoo.com (S.M.); 4 Department of Immunology, Semnan University of Medical Sciences, Semnan 35147-99442, Iran; dhaghmorad@gmail.com; 5 Department of Clinical Science, University of Bergen, 5020 Bergen, Norway; 6 Department of Clinical and Molecular Medicine, Norwegian University of Science and Technology (NTNU), 7028 Trondheim, Norway; 7 Department of Biosciences and Nutrition, Karolinska Institutet, 14183 Huddinge, Sweden; 8 Cancer Research Center, Faculty of Medicine, Semnan University of Medical Sciences, Semnan 35147-99442, Iran; 9 Department of Bacteriology and Virology, Faculty of Medicine, Semnan University of Medical Sciences, Semnan 35147-99442, Iran

**Keywords:** microbiome, dysbiosis, next-generation sequencing, metagenomics, metabolomics

## Abstract

The gut microbiota significantly impacts human health, influencing metabolism, immunological responses, and disease prevention. Dysbiosis, or microbial imbalance, is linked to various diseases, including cancer. It is crucial to preserve a healthy microbiome since pathogenic bacteria, such as *Escherichia coli* and *Fusobacterium nucleatum*, can cause inflammation and cancer. These pathways can lead to the formation of tumors. Recent advancements in high-throughput sequencing, metagenomics, and machine learning have revolutionized our understanding of the role of gut microbiota in cancer risk prediction. Early detection is made easier by machine learning algorithms that improve the categorization of cancer kinds based on microbiological data. Additionally, the investigation of the microbiome has been transformed by next-generation sequencing (NGS), which has made it possible to fully profile both cultivable and non-cultivable bacteria and to understand their roles in connection with cancer. Among the uses of NGS are the detection of microbial fingerprints connected to treatment results and the investigation of metabolic pathways implicated in the development of cancer. The combination of NGS with machine learning opens up new possibilities for creating customized medicine by enabling the development of diagnostic tools and treatments that are specific to each patient’s microbiome profile, even in the face of obstacles like data complexity. Multi-omics studies reveal microbial interactions, biomarkers for cancer detection, and gut microbiota’s impact on cancer progression, underscoring the need for further research on microbiome-based cancer prevention and therapy.

## 1. Introduction

### Using New Technologies to Analyze Gut Microbiota

The human microbiome is a diverse ecosystem comprising bacteria, fungi, and viruses and includes, in total, approximately 10^14^ microorganisms. Under optimal conditions, these microbes maintain a symbiotic relationship with their host, especially within the gastrointestinal tract [1,2]. Each microbial species possesses significant metabolic activities that are essential for human health. For example, it has been demonstrated that *Bifidobacterium longum* induces the secretion of pro-inflammatory cytokines such as tumor necrosis factor (TNF)-α and interleukin (IL)-10, which may shield the host against the development of tumors [3]. However, dysbiosis—alterations in microbiota composition—has been linked to various health issues, including allergies, Type 2 diabetes, obesity, and colorectal cancer (CRC). Intestinal fungal dysbiosis, caused primarily by yeast species from genera *Candida*, *Malassezia*, and *Saccharomyces*, is associated with diseases such as alcohol-related liver disease [4], hepatitis B [5], colon cancer [6], autistic spectrum disorders [7], Parkinson’s disease [8], and inflammatory bowel disease (IBD), According to some findings, dysbiosis also contribute to the onset of Crohn’s disease [9,10].

Recent research has identified several molecular pathways through which pathogenic bacteria contribute to tumor development. For instance, *Escherichia coli* (pks+) produces colibactin, a genotoxin that induces DNA alkylation, which can lead to colon cancer [11,12]. Other well-known tumor-promoting bacteria, including *Fusobacterium nucleatum* and *Helicobacter pylori*, may cause malignancy by a variety of complex mechanisms, such as chronic inflammation, degradation of DNA, and the activation of oncogenic pathways. Therefore, maintaining both the composition and functionality of the gut microbiome is crucial for overall host health. To better understand the distinct characteristics of healthy and pathological microbiomes and their relationship to disease, researchers are employing advanced methodologies [13]. Over the last two decades, significant progress in microbiome research has been driven by technological breakthroughs, enabling the analysis of vast datasets without relying solely on conventional culture techniques [13]. Early studies identified microbial diversity through microscopy and isolation, but challenges in cultivation limited the understanding of this diversity. Initial techniques, such as terminal-restriction fragment length polymorphism (T-RFLP), denaturing gradient gel electrophoresis (DGGE), and temperature gradient gel electrophoresis (TGGE), revealed variations in microbial populations but lacked detailed taxonomic information [14].

To deepen our understanding of gut microbiota-host interactions, innovative approaches combining biology and engineering are necessary. PCR-based high-throughput sequencing has significantly improved microbiome analysis, detecting microorganisms in complex communities and paving the way for next-generation sequencing technologies. Several cutting-edge technologies are currently utilized in gut microbiota research, including Next-Generation Sequencing (NGS), metagenomics, metatranscriptomics, metabolomics, multiomics, fecal microbiota transplantation (FMT), and advanced culturing techniques. High-throughput sequencing methods commonly used in microbiome research include PCR amplicon-based sequencing (such as 16S rRNA, 18S rRNA, and internal transcribed spacer [ITS] sequencing), DNA-based shotgun metagenomic sequencing, RNA-based metatranscriptomic sequencing, and viromic sequencing [15]. The 16S rRNA gene is widely used for studying bacterial populations, while ITS sequencing is specifically employed for identifying fungi. Shotgun metagenomic sequencing provides a primer-independent approach, reducing biases commonly associated with amplicon-based methods. Metagenomics offers insights into the DNA composition of microbial communities; however, it has limitations in assessing gene expression. Metatranscriptomic sequencing addresses this gap by enabling the detection of active organisms and assessing effectively transcribed genes, providing a more comprehensive understanding of microbiota functionality. Additionally, metaproteomics and metabolomics provide insights into the functional pathways of gut microbes [16]. This review explores recent advancements in microbiome research and their potential applications in cancer detection, prognosis, and treatment.

Scientific attention has been directed toward finding microbiome-based biomarkers that help improve diagnoses, prognostics, and treatment approaches as a result of a growing body of research over the past decade that has demonstrated the critical involvement of the human microbiome in several diseases. The high-dimensional, compositional structure of the data, its intrinsic variability, and the difficulty of combining microbiome data with other biological datasets make the analysis of microbiome data increasingly complex as the field develops. One particularly useful tool for navigating these complications is machine learning (ML), which provides pathways for analyzing and utilizing large microbiome datasets. But to take advantage of this potential, specialists in ML and microbiome research must work closely together, with a strong foundation in interdisciplinary communication and established methodologies. While the International Human Microbiome Coordination and Support Action focuses on innovation, making sure that science is rigorous and reproducible across contexts, noteworthy initiatives like the ELIXIR Machine Learning Focus Group and the creation of ISO standards are promoting structured ML applications. Following the FAIR principles—making sure machine learning tools for microbiome analysis are Findable, Accessible, Interoperable, and Reproducible—is essential to furthering microbiome research. These guidelines promote the creation of standardized procedures, which are particularly important in clinical settings because accessibility and repeatability have a significant influence on nutrition and health interventions [17].

**Figure 1:**
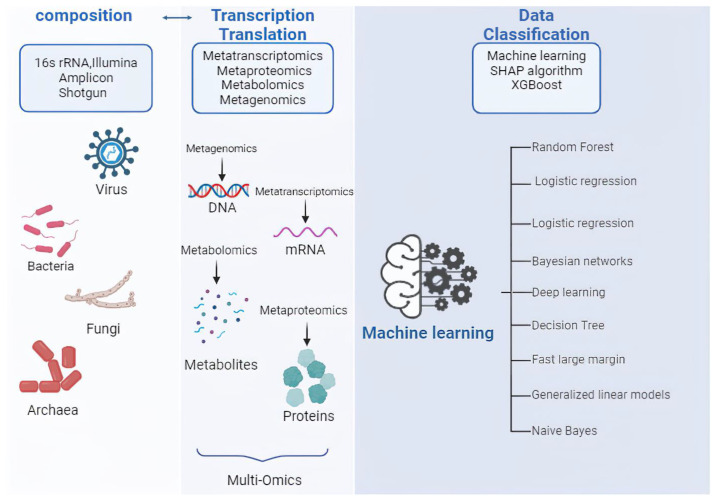
A visual representation of the multi-omics workflow, including DNA, RNA, protein, and metabolite analysis with icons for sequencing, mass spectrometry, and machine learning (**Left**). Examples of machine learning algorithms in metagenomics data classification (**Right**). SHAP (a mathematical method used to explain the predictions of machine learning models) and XG Boost (eXtreme Gradient Boosting). The figure was designed using BioRender software. To generate the Figure, Biorender.com was used (accessed on 1 November 2024).

It is becoming increasingly difficult to translate microbiome research into clinical applications, particularly when it comes to diagnosis, prognosis, and therapy response monitoring. It is crucial to improve bioinformatics techniques, such as increasing species identification precision and combining microbiome data with other omics data. These developments could expand the therapeutic value of microbiome results by enabling prediction models and establishing causal links. However, using machine learning to analyze microbiome data is still challenging because no single analytical method can provide insights that are generally applicable. As a result, integrating various machine learning tools, including cutting-edge techniques, e.g., deep learning, may reveal complex patterns in microbial populations, providing a better understanding of how microbes affect health and illness. However, there are drawbacks to this integrative method as well, including issues with sample variety, model interpretability, and ethical considerations. A significant answer to the demand for transparency in machine learning models, particularly in healthcare applications where physicians need comprehensible and trustworthy explanations for model-based decisions, is explainable artificial intelligence (XAI). The need for diverse datasets and strong frameworks for informed consent is further highlighted by ethical considerations, including data privacy and the possibility of bias [17]. (Table 1 and Figure 1).

## 2. The Use of Technology to Predict Cancer via Gut Microbiota

The relationship between gut microbiota and cancer prognosis is a developing field driven by advances in artificial intelligence and microbiome analysis technologies. Next-generation sequencing (NGS), in particular, has revolutionized cancer research by enabling sensitive detection of microbial DNA, offering valuable insights into the complex interactions between the microbiome and carcinogenesis. However, these methods come with drawbacks as well [27]. For example, it can be difficult to differentiate between real microbiome data and contaminant DNA, which is particularly important in samples with low microbial biomass. The issues presented by the large amounts of data generated from high-throughput sequencing are mostly addressed by artificial intelligence (AI) and, in particular, machine learning (ML). The categorization of illnesses linked to certain microbiome profiles has been made possible through the analysis of microbial data using ML algorithms [28]. For instance, certain cancer types have been successfully classified based on microbiological data using logistic regression, random forest, support vector machines, gradient boosting, and deep learning models [29]. These models’ remarkable accuracy rates demonstrate how ML might improve diagnostic skills and aid in the early diagnosis of cancer [30]. In a recent study, researchers developed supervised ML models to distinguish between various cancer types based on microbial profiles using data from The Cancer Microbiome Atlas (TCMA). A thorough data collection and pre-processing step was required for the study to guarantee that only relevant genera were examined. The integrity of the study process was strengthened by carefully addressing ethical issues related to data access. By using the inherent feature selection capabilities of random forests to find pertinent microbial taxa, the experimental design highlighted the significance of feature selection. Furthermore, to maximize model performance, sophisticated methods like dimensionality reduction and oversampling were used, highlighting the intricacy of modern approaches to microbiome research [31].

The functional capacities of microbial communities underscore the broader significance of microbiome research in cancer. The diverse microbes that make up the gut microbiome play essential roles in maintaining host health, including the metabolism of food components, immune system education, and pathogen defense. These microbial activities have a significant impact on a range of health outcomes, including an individual’s vulnerability to certain illnesses. Understanding the functional properties and composition of microbes is particularly critical in cancer research, such as dysbiosis—an imbalance in microbial communities—has been linked to tumor development. High-throughput techniques, such as shotgun metagenomics and amplicon sequencing, have facilitated complete microbial community profiling. These methods not only improve microbial diversity characterization but also enable predictions of functional activities within the microbiome. For example, metagenome predictions based on amplicon sequencing data can be generated by researchers using methods such as PICRUSt, which identifies enriched pathways linked to inflammation in cancer tissues. Our knowledge of the microbiome´s role in cancer development and the response to therapy is further improved by the combination of metagenomics, meta-transcriptomics, and metabolomics. This process also reveals possible biomarkers for early diagnosis and treatment approaches [32]. Studies reveal that specific microbial profiles can serve as markers for cancer development and therapeutic effectiveness. For instance, research has demonstrated a correlation between certain microbial components and cancer therapy outcomes. Identifying these microbial indicators facilitates the development of personalized treatment plans that account for the patient’s microbiome composition, in addition to helping to understand the pathogenesis of cancer. Furthermore, there are therapeutic implications to the correlation between the microbiota and cancer that go beyond simple prediction. Studies investigating how microbial profiles may influence therapeutic sensitivity or resistance to various therapies, including chemotherapy and immune checkpoint inhibitors, are underway to examine the impact of the gut microbiome on cancer treatment results. Gaining insight into these processes may help develop novel approaches to modifying the gut microbiota to improve patient outcomes and treatment efficacy. The potential for creating cutting-edge diagnostic techniques and treatment strategies is becoming increasingly evident as research on the connection between the microbiome and cancer continues to uncover its complexity. The intricate relationship between host health and microbiota underscores the importance of a multidisciplinary strategy integrating genetics, computational analytics, and microbiology to enhance cancer research and patient treatment [33]. In this review article, we tried to examine the different techniques and methods of gut microbiota analysis. We divided these techniques into four sections, which include (1) Sequencing Platforms (NGS), (2) Bioinformatic Data Analysis Methods (Meta-omics, Operating taxonomic unit (OTU), amplicon sequence variant (ASV), (3) Machine Learning Techniques (Support Vector Machine (SVM) and SHAP algorithm), and (4) Emerging Technologies (Multi-Omics, Cutting-Edge Technology, Amplicon Sequencing, 16S rRNA Gene Sequencing, and Mapping Cancer’s Path).

### 2.1. Sequencing Platforms

#### Next-Generation Sequencing (NGS)

NGS has revolutionized the study of gut microbiota by offering thorough insights into the microbial composition and possible roles of these diverse communities. Technologies such as metagenomics, whole-genome sequencing, and 16S rRNA gene sequencing enable an in-depth analysis of microbial diversity, even in challenging environments like the human gut. NGS plays a critical role in elucidating the complex relationship between gut microbiota and carcinogenesis, progression, and treatment outcomes in cancer research. Additionally, NGS facilitates the investigation of microbial metabolic pathways and gene activities, shedding light on the potential role of microbiome in cancer development. By examining the functional capabilities of gut microbiota, researchers can identify microbial genes involved in key processes such as inflammation, DNA repair, and metabolism, processes that are integral to cancer development. Complete functional analysis, made possible by NGS, is essential for understanding the several mechanisms by which the microbiota either stimulates or inhibits cancer. One of NGS’s key advantages is its ability to process large datasets, especially given the immense diversity of the gut microbiota. However, sophisticated bioinformatics tools are necessary to efficiently process and interpret the vast amount of data generated by NGS, allowing for the reliable identification of key microbial players and their functional roles. Thus, NGS provides not only a detailed view of microbial diversity but also insights into the functional significance of these communities in cancer development [34,35].

Moreover, unusual microbial species that traditional sequencing techniques can miss can be found using NGS’s sensitivity. Despite their low prevalence, these uncommon taxa may be important players in processes connected to cancer, either directly interacting with host cells or indirectly affecting the general makeup and functionality of the microbiota. Therefore, NGS offers a thorough and detailed understanding of the gut microbiota’s role in cancer, which is crucial for developing medicines that target the microbiome. To sum up, NGS is an essential tool for gut microbiota research, especially when it comes to cancer. It is a potent technique for examining the connection between gut microbiota and cancer because of its capability to handle big, complicated datasets and its ability to give in-depth taxonomic and functional insights into the microbiota. Through the use of NGS technology, scientists can identify important microbial signatures and mechanisms that could guide the creation of new cancer diagnostic instruments and treatment plans. Personalized medicine techniques based on microbial profiling and our understanding of the function of the microbiota in cancer can both be greatly advanced by the incorporation of NGS into cancer research [36].

The question of whether a single microbial profile can be used to categorize many cancer types remains unanswered despite the fact that prior studies mostly focused on using separate microbial signatures to distinguish between various tumors. Given the complexity of the microbiome, machine learning models are useful because they are excellent at identifying subtle trends that may escape traditional research [31]. Applications of ML in healthcare have surpassed cancer to encompass genetic analysis, imaging, and electronic health record administration. These methods have been applied mainly to colorectal cancer in microbiome research, but newer studies are starting to investigate other cancer types as well, potentially identifying biomarkers for treatment response and early diagnosis. The methods used to characterize the microbiome mostly depend on nucleic acid analysis, and they include a range of sample types, including blood, tissue biopsies, mucosal swabs, and feces. Different sample types provide unique insights into the interactions between the microbiome and the host, highlighting the importance of context in sample selection for research. Contamination is still a serious issue, though, as cross-contamination and external DNA might distort the outcomes [31].

Although taxonomic makeup is important in microbiome research, a thorough understanding of its functional characteristics is just as important. For the most part, taxonomic profiles are used in machine learning applications today. However, there is growing evidence that including functional data might improve prediction accuracy. The utilization of next-generation sequencing technology facilitates high-throughput functional profiling, which opens up new avenues for investigating the relationship between various data types and how they relate to cancer detection. The use of ML in cancer applications appears to have a bright future despite the difficulties associated with microbiome data, notably the high frequency of zero values and unpredictability. It will be crucial to create models that can handle these complexities to find microbial signatures associated with particular cancer features while excluding connections that might be confusing. Additionally, integrating ML models into clinical settings calls for careful consideration of patient privacy, ethical issues, and the necessity of communicating clearly about the limitations of microbial biomarkers in cancer diagnosis [37].

NGS encompasses a broad range of platforms that have revolutionized microbiome studies by enabling a deeper understanding of microbial diversity and function. Among these platforms, Ion Torrent and Illumina are prominent due to their high accuracy and cost-effectiveness. Illumina sequencing-by-synthesis method produces short but highly accurate reads, which makes it perfect for microbial diversity and metagenomic research. Ion Torrent, on the other hand, uses ion-sensitive devices to chemically detect nucleotide incorporation, providing a simpler and faster sequencing procedure but with slightly lower accuracy in certain applications [34]. In addition to these short-read platforms, long-read sequencing technologies like PacBio and Oxford Nanopore are increasingly utilized for more advanced microbiome research. PacBio, through its single-molecule real-time (SMRT) sequencing, provides long and highly accurate reads, enabling the complete reconstruction of microbial genomes and detailed analysis of epigenetic modifications. Oxford Nanopore, with its real-time sequencing capability and portable devices, is highly flexible for environmental sampling and rapid microbial diagnostics. Its long-read capacity is particularly beneficial for identifying structural variants and resolving genomic repeats [38].

Emerging technologies such as Element Biosciences and Singular Genomics are also transforming the field by aiming to enhance sequencing accuracy, speed, and cost-efficiency. While Singular Genomics offers flexible and user-friendly systems to fulfill a range of research objectives, including microbiome and personalized medicine applications, Element Biosciences concentrates on lowering sequencing costs while improving data quality. The integration of these diverse technologies offers a powerful solution to overcome the limitations of individual platforms [39]. For instance, microbial genome reconstruction may be greatly enhanced, and a more thorough examination of microbial ecosystems can be obtained by fusing the long-read and structural resolution capabilities of PacBio or Oxford Nanopore with the high accuracy and cost-effectiveness of Illumina. Furthermore, by enabling a wider spectrum of academics to use advanced sequencing methods, the adoption of cutting-edge platforms like Element Biosciences and Singular Genomics may speed up research. In addition to improving data clarity, these multi-technology methods make it possible to find intricate host-microbiome interactions. These developments make it easier to profile antibiotic resistance, identify microbial makeup more precisely, and even create individualized treatment plans for medical research. As a result, a key component of current microbiome research is the integration and use of various sequencing technologies, which advances our understanding and improves human health outcomes [38].

## 3. Bioinformatics Data Analysis Methods

### 3.1. Meta-Omics

Meta-omics technologies are increasingly employed to explore the gut microbiome’s involvement in cancer, offering insights into its genetic composition and metabolic functions. Methods like shotgun metagenomics and metabolomics have identified specific bacterial species associated with colorectal cancer, underscoring the microbiome’s potential role in oncogenesis. The human intestine is a complex ecosystem comprising host cells, microbial communities, and various molecules. To capture the functional dynamics of this ecosystem, metatranscriptomics and metaproteomics measure RNA transcripts and proteins, providing a detailed snapshot of the gut microbiome’s active processes and interactions with the host. Shotgun metagenomics is a particularly comprehensive tool in microbiome analysis, examining all DNA fragments within a sample to yield precise information on microbial species abundance, taxonomic composition, and functional potential. This technique also enables researchers to reconstruct complete microbial genomes, enhancing taxonomic and functional insights. However, its focus remains primarily on the microbiome’s genetic potential, which does not always mirror its actual activity. Integrating metagenomics with metatranscriptomics addresses this limitation by coupling genetic potential with data on gene expression, offering a more nuanced understanding of the gut microbiome’s role in health and disease. This synergy between metagenomics and metatranscriptomics thus provides a valuable, multi-dimensional view of the microbiome’s functionality, which is particularly relevant for studying its role in cancer biology. Metagenomics provides insights into the host-microbiome interactions that may enhance susceptibility to cancer by revealing differences in microbial diversity and identifying oncogenic or protective microbial species [40]. Changes in metabolites, including bile acids or SCFAs, have been linked to an increased risk of cancer via influencing pathways leading to cellular proliferation or inflammation. Furthermore, by detecting variations in the microbial composition or metabolic profiles prior to the onset of clinical signs, meta-omics can aid in the early identification of cancer. A systems biology viewpoint is formed by integrating various omics technologies, which makes it possible to forecast cancer risk more precisely based on microbial-host interactions, environmental variables, and metabolic alterations. In the end, meta-omics is opening the door to early treatments and individualized preventative plans catered to each person’s own microbial and metabolic profile [41,42].

### 3.2. Operating Taxonomic Unit and Amplicon Sequence Variant

Operating taxonomic unit (OTU) and amplicon sequence variant (ASV) are foundational concepts in microbiome research, each serving as a strategy for grouping microbial sequences but differing in their levels of precision and approach. OTUs, the traditional method, group sequences based on a fixed similarity threshold. This threshold-based clustering considers sequences that fall within this similarity range as representing the same taxonomic group, often approximating species-level distinctions. However, because OTUs rely on arbitrary clustering thresholds, they may overlook subtle sequence differences, sometimes grouping genetically distinct organisms together if their sequences are sufficiently similar. Despite this limitation, OTUs have been widely used for their simplicity and efficiency, making them suitable for broader taxonomic studies where exact sequence distinctions are less critical [43].

In contrast, ASVs represent an advancement in microbial sequence analysis, eliminating the reliance on similarity thresholds and instead identifying unique sequences at the single-nucleotide level. This higher-resolution approach allows researchers to detect true biological variations with greater precision. ASVs do not cluster sequences but rather identify and distinguish every unique sequence variant, providing a reproducible and consistent representation of microbial diversity across datasets. This characteristic of ASVs is particularly valuable when comparing studies, as it enables consistent identification of microbial taxa independent of arbitrary clustering parameters. By capturing true biological diversity, ASVs enhance the ability to track fine-scale variations within microbial communities, making them especially relevant in studies focused on ecological or functional differences within closely related microbial populations. Consequently, while OTUs offer a broader, more approximate view of microbial taxonomy, ASVs provide the granularity necessary for detailed analyses of microbial diversity and are increasingly adopted as a standard in modern microbiome research [44].

The OTU clustering algorithm is generally categorized into two methods: reference-based and de novo. Reference-based algorithms, such as Mothur, VSEARCH, and USEARCH, align reads with reference databases and subsequently cluster the reads based on alignment results [45]. On the other hand, de novo algorithms, including Swarm, CD-HIT, and UPARSE, directly cluster OTUs based on the similarity of short DNA reads without requiring a reference database. Following the sequencing and classification of microbiota data, bioinformatics tools such as QIIME, PICRUSt, STAMP, CLARK, LefSe, Mothur, and Kraken, along with artificial intelligence methods, are employed to analyze microbial characteristics and functions. These tools facilitate tasks such as variation analysis, differential abundance analysis, and classification. A critical application of these techniques is the identification of microbial markers that can be used to predict cancer. With the rapid advancements in bioinformatics, numerous databases have been established to compile microbiota data from diverse regions. These databases classify microbiota based on phenotypes, age, gender, geographic location, and body mass index [46].

One of the key advantages of these resources is their ability to enable comparative analysis of intestinal microbiota in cancer patients from various countries, providing insights into the microbial profiles associated with different types of cancer [47]. Traditional methods of classifying comparable sequences based on a predefined similarity threshold, or OTUs, provide valuable information about the composition and diversity of microorganisms in a given sample. By analyzing ASVs, researchers can identify specific microbial signatures associated with different cancer types, thus improving the ability to stratify an individual’s cancer risk. The prognostic potential of microbiome research in cancer is enhanced by incorporating both OTUs and ASVs in microbial community profiling. For instance, ASV analysis can reveal variations in microbial populations that are linked to metabolic pathways involved in carcinogenesis, including immune regulation and inflammation. These insights help to establish a clearer connection between microbiome and cancer development. By utilizing both OTUs and ASVs, a more comprehensive understanding of the microbiome’s role in the etiology of cancer can be achieved. This, in turn, could pave the way for the development of personalized preventative measures and targeted therapeutic strategies, ultimately improving cancer management [46].

## 4. Machine Learning Techniques

### 4.1. Support Vector Machine (SVM)

The application of modern machine learning (ML) technologies, including Support Vector Machines (SVMs), has significantly improved predictive capabilities in cancer diagnosis and prognosis, particularly in studies involving gut microbiota. Early and accurate cancer diagnosis is critical, as it directly shapes subsequent clinical care plans and improves patient outcomes. SVMs, as a powerful ML approach, extract relevant features from extensive, complex datasets, which facilitates the development of highly effective prediction models. By efficiently identifying distinguishing patterns, recent research has demonstrated the efficacy of SVMs in differentiating various cancer types, thus highlighting their potential to advance precision diagnostics and personalized cancer treatment strategies [48]. One noteworthy study, for example, used ML to evaluate DNA methylation patterns and was able to discriminate between head and neck metastases and initial lung squamous cell carcinomas. To further validate the prediction potential of the model, another research effort used SVMs to discover particular genes associated with vascular invasion in hepatocellular carcinoma. As a complete gene expression data library, the Gene Expression Omnibus (GEO) was created in 2000 by the National Center for Biotechnology Information (NCBI). This database was used by researchers to do a thorough investigation of the gene expression patterns associated with duodenal cancer and familial adenomatous polyposis (FAP), discovering differentially expressed genes that differentiate cancer cases from healthy circumstances. A cancer risk prediction system for patients with FAP was created using SVM-based modeling, illuminating the usefulness of ML in oncology [49].

Moreover, these differentially expressed genes were successfully utilized in the building of SVM classifiers, which helped to classify data. Recursive feature elimination was used in this approach to fine-tune the selection of ideal genes, guaranteeing the predicted accuracy and resilience of the model. Researchers evaluated patients’ risk of developing cancer by verifying the classifier with separate datasets, highlighting the potential of ML to support clinical decision-making. Within the healthcare domain, the use of ML techniques such as SVMs facilitates personalized medicine by enabling customized treatment plans that are determined by an individual’s genetic, demographic, and clinical characteristics. SVMs also aid in risk stratification, allowing medical professionals to group patients according to their propensity to acquire particular ailments. In the field of cancer genomics, where developing new biomarkers and understanding the genetic causes of cancer is essential to improving treatment approaches, this kind of stratification is highly relevant. Using SVMs has significant implications for many areas of healthcare, such as gene expression analysis, tailored treatment strategies, and predictive modeling for patient outcomes. In this context, the identification of crucial genes linked to diseases, e.g., leukemia and colon cancer, is facilitated by the use of microarray data. SVMs have also shown promise in medical imaging, where they assist with image analysis-based diagnosis. The increasing reliance on data-driven strategies and the growing availability of electronic health records highlight how critical ML is to enhancing clinical outcomes as the healthcare landscape evolves. By streamlining the decision-making process and improving diagnostic accuracy, this technological integration ultimately improves patient care [48,50].

### 4.2. SHAP Algorithm

A key component of the eXplainable Artificial Intelligence (XAI) architecture is the SHAP (SHapley Additive exPlanations) algorithm, which is especially useful when attempting to comprehend the intricate relationship between gut microbiota and cancer prevention. Scientists can clarify the roles played by several variables in the classification of control and colorectal cancer (CRC) samples using this model-agnostic post-hoc explanation approach. The SHAP method, which draws on concepts from cooperative game theory, creates comprehensible linear models for individual cases, clearly illustrating the contributions of each feature to the final prediction [51]. Calculating the differences in model predictions when specific characteristics are present versus when they are not is the fundamental computational step of SHAP values. To ensure accuracy, this thorough analysis requires evaluating every possible subset of features, which means retraining the model on these subsets. More specifically, the SHAP value provides a detailed view of the relevance of a given feature in an instance by aggregating its contributions over all possible feature combinations [52].

Utilizing SHAP in our investigation of CRC allows us to uncover significant microbial markers associated with cancer risk. For example, *Granulicatella* and *Porphyromonas* were identified by the algorithm to be important genera for distinguishing between colorectal cancer subtypes, which is consistent with findings from other studies that highlight their involvement in tumor growth. By applying SHAP, our model becomes more transparent and allows for a better comprehension of the ways in which different microbial populations influence the development of cancer. It is crucial to recognize some of the limits of our study, even if SHAP analysis has a lot to offer in terms of interpretability. Our findings cannot be applied broadly because of the small sample size and single-center data gathering. To strengthen SHAP analyses’ robustness and conclusions’ external validity, more extensive, multi-center investigations should be the focus of future studies. Further research should address this restriction in order to validate the SHAP results and improve their application in clinical practice. Another disadvantage of the study is the lack of experimental validation [53].

## 5. Emerging Technologies

### 5.1. Amplicon Sequencing

Amplicon sequencing, a specialized form of NGS, offers significant advantages over older techniques. NGS is faster, generates richer datasets, and can effectively analyze a wide variety of sample types, making it an indispensable tool for unraveling the complexities of the gut microbiome. In microbiome research, amplicon sequencing is particularly valuable due to its reliance on marker genes, specific genes that serve as unique identifiers for different microbes within a sample. These marker genes function like fingerprints for microbes, containing conserved regions that are similar across many species, indicating microbial origin, as well as variable regions that are unique to each species, providing a distinct identification code. The 16S rRNA gene is the most commonly used marker for identifying prokaryotic microbes. Its conserved and variable regions strike a crucial balance, allowing researchers to distinguish bacterial communities accurately and efficiently. For eukaryotes, genes like the 18S rRNA and the internal transcribed spacer (ITS) region play a similar role, offering effective identification of eukaryotic species within a sample. Selecting the appropriate marker gene depends on the specific research focus and the target organisms, enabling scientists to capture a detailed microbial profile relevant to their study goals. This versatility makes amplicon sequencing a cornerstone in advancing our understanding of microbial diversity and its implications for health and disease [54].

The highly targeted NGS method known as amplicon sequencing has important applications in cancer risk prediction. It enables the examination of specific genomic regions that have been linked to an increased risk of developing cancer, especially those located in genes associated with hereditary malignancies such as TP53, BRCA1, and BRCA2. Amplicon sequencing can identify germline mutations or somatic alterations that raise the risk of developing cancer by focusing on predefined regions of interest. The ability of amplicon sequencing to detect low-frequency variants, such as single nucleotide polymorphisms (SNPs), insertions, deletions, and copy number variations in oncogenes and tumor suppressor genes, is a significant benefit in predicting cancer risk. This allows medical professionals to identify individuals who are at greater risk due to a genetic predisposition [55]. One of the most common applications of amplicon sequencing is the analysis of the 16S ribosomal RNA (rRNA) gene, a highly conserved region in prokaryotic genomes. This gene contains variable regions that allow for the identification and classification of bacteria and archaea [56].

#### 16S rRNA Gene Sequencing

With the development of DNA sequencing technology, the capacity for assessing the composition of diverse microbial communities has improved, enabling more accurate and faster identification of individuals within those communities based on their taxonomic classification [56]. Previous sequencing technologies showed a slow pace and required significant expenses. The process of sequencing analysis of the 16S rRNA gene previously involved cloning the whole gene onto plasmid vectors, introducing it into suitable hosts, and subsequently sequencing it. Currently, the most frequently used approach to analyze the construction of the gut microbiome is to extract all DNA from the samples, amplify specific regions within the highly similar 16S/18S rRNA genes using PCR, and then sequence those amplified fragments using high-throughput techniques, e.g., with Illumina sequencing. This strategy has bypassed the necessity of cloning individual genes, conducting blotting for specific RNA, or cultivating individual bacteria to figure out the various parts of a community. This is the procedure followed for performing assignments and duties [57].

The technique involves extracting microbial DNA from a habitat using specialized procedures, such as PCR and quantitative PCR, to amplify the target area of the 16S rRNA gene. The DNA is then processed to create a collection of genetic material suitable for high-throughput sequencing. Advanced NGS systems, such as Illumina sequencing, produce millions of short DNA sequences. The sequences are then analyzed using bioinformatics programs, which filter high-quality data, fix errors, and categorize sequences into specific microbial categories using reference databases. This helps identify microbial taxonomic elements in the original sample and assess the quantity and diversity of microbial species. This study provides valuable information on the composition and organization of the microbial community, allowing researchers to make decisions on the diversity of microorganisms and their environmental importance [58]. Although the 16S rRNA gene has both positive and negative effects, it is widely recognized as the predominant genetic marker for cleaning applications. The main reason for this is its enormous frequency in almost every bacteria, its enduring functionality unchanged by mutations or evolution, and its notable size of 1500 base pairs for information processing [15]. 16S rRNA gene sequencing is an essential method for studying the gut microbiome, providing crucial insights into its structure, genetic diversity, and potential impact on health and disease, including cancer [59].

Because 16S rRNA gene sequencing provides insight into the variety and makeup of the gut microbiome, it is a vital tool for predicting cancer risk. Systemic inflammation, immunological responses, and metabolic processes are all impacted by changes in microbial populations and are all connected to the development of cancer. For example, colon cancer has been linked to certain bacterial taxa that are linked to a pro-inflammatory state. Researchers can find certain microbial fingerprints that act as biomarkers for cancer susceptibility by examining the 16S rRNA gene. Early risk assessment is made easier by this technology, which allows for the identification of changes in microbial populations linked to food patterns, lifestyle variables, and genetic predispositions. Additionally, by comprehending these microbial interactions, specific therapies to reduce the risk of cancer may be developed, such as dietary changes or probiotics. Therefore, 16S rRNA gene sequencing is a promising method for personalized treatment and cancer prevention [59].

### 5.2. Mapping Cancer’s Path

The term “Mapping Cancer’s Path” highlights the critical role of understanding microbial influences in the complex processes underlying cancer development. This involves methodically determining the way in which certain infections and gut bacteria contribute to carcinogenesis. These contributions are frequently mediated by processes that collectively disturb cellular homeostasis and promote malignant transformation, including immune evasion, DNA damage, and chronic inflammation. Research in this area focuses on elucidating how dysbiosis—imbalances in the gut microbiota—can trigger or worsen oncogenic pathways. Some bacteria, for example, can create genotoxins that directly damage DNA, while others can change the immune environment in the area, making it possible for tumor cells to evade immune surveillance. Moreover, persistent inflammation induced by pathogenic microorganisms can create a pro-tumorigenic environment, fostering cellular proliferation and mutation.

#### 5.2.1. Gastrointestinal Cancers and Microbiota

The gut microbiota is crucial to the development of cancer, especially CRC, the most prevalent disease linked to dysbiosis [60]. Microbiota-related influences are also seen in other cancers, including lung, breast, and liver tumors.

- *Helicobacter pylori* is strongly linked to stomach cancer. As a class 1 carcinogen, it disrupts the stomach lining and produces H2O2, which triggers programmed cell death and DNA damage. This increases apoptosis resistance, leaving infected cells more susceptible to malignant transformation. Moreover, *H. pylori* enhances PD-L1 expression on stomach cells, allowing immune evasion and progression toward cancer [61];
- *Escherichia coli* produces colibactin, a genotoxin that induces double-strand DNA breaks and chromosomal instability. This bacterium also generates reactive oxygen species (ROS), exacerbating DNA damage and disrupting the cell cycle, creating an environment conducive to CRC [62];
- *Bacteroides fragilis*, particularly the enterotoxigenic subtype (ETBF), secretes the BF toxin, which promotes inflammation by increasing COX-2 and releasing prostaglandin E2 (PGE2). This activates STAT3 signaling, compromising cellular defenses by degrading E-cadherin and increasing DNA damage. Additionally, ETBF’s fragilysin triggers precancerous inflammatory cascades, accelerating tumorigenesis [63];
- *Fusobacterium nucleatum* contributes to CRC via inflammatory pathways such as TLR4-NF-κB signaling and abnormal methylation of tumor suppressor genes (e.g., *CDX2, MLH1*). Its adhesion protein, *FadA*, disrupts the E-cadherin/β-catenin pathway, driving uncontrolled cell proliferation [64].

#### 5.2.2. Microbial Mechanisms in Non-Gastrointestinal Cancers

Microbiota’s impact extends beyond the gastrointestinal tract, influencing cancers such as liver, breast, and lung malignancies.

- *Epstein-Barr* virus (EBV), associated with mononucleosis, contributes to cancers like lymphomas, gastric cancer, and nasopharyngeal carcinoma. It enhances immune evasion by upregulating PD-L1 on cancer cells through viral microRNAs, silencing FOXP1 and PBRM1, and inhibiting NK cell functions [65,66];
- In breast cancer, bacterial families such as *Faecalibacterium, Ruminococcaceae*, and *Clostridia* disrupt cellular scaffolding, facilitating metastasis. Research has shown that gut microbiota can interfere with cytoskeletal dynamics, enhancing the metastatic potential of breast cancer cells [67];
- The gut–liver axis links gut bacteria to liver cancer through metabolites such as deoxycholic acid (DCA) and lipoteichoic acid (LTA), which induce chronic inflammation and damage hepatocytes [68,69].

#### 5.2.3. Multi-Omics in Mapping Cancer’s Path

Recent advancements in multi-omics approaches, including metagenomics, transcriptomics, and metabolomics, have revolutionized cancer research. Studies have identified bacterial signatures, such as elevated levels of *Actinobacteria* and *Bifidobacterium* in lung cancer patients, which could serve as diagnostic markers. Multi-omics methodologies enable comprehensive mapping of the interplay between microbes and their genetic, functional, and host interactions, revealing critical pathways in cancer progression [70].

**Figure 2:**
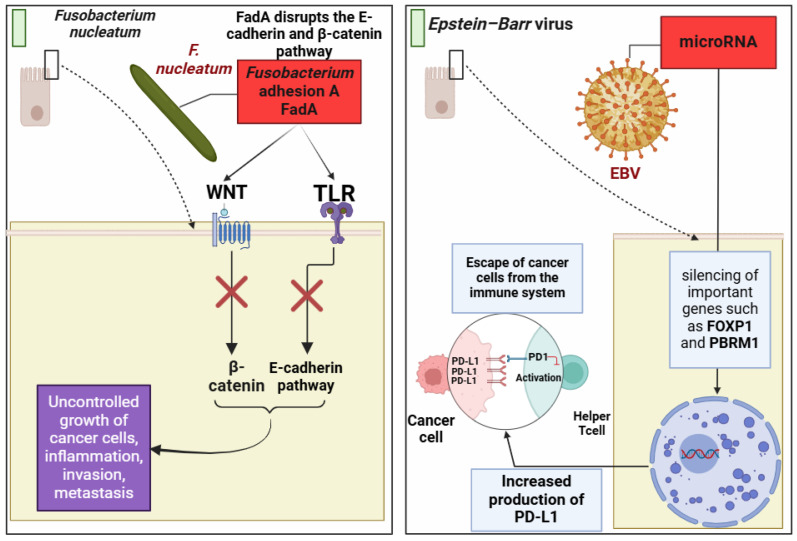
An illustration of how *Epstein-Barr* virus (EBV) and *Fusobacterium nucleatum* contribute to colorectal cancer development and immunological evasion. *F. nucleatum* stimulates inflammatory pathways in colon epithelial cells and is linked to gene methylation that targets anti-cancer genes like MLH1 and CDX2. It also interferes with the Wnt/β-catenin pathway through its adhesion protein FadA, leading to uncontrolled cell proliferation and an increased cancer risk. EBV-induced mononucleosis, linked to deadly malignancies, upregulates the production of the PD-L1 protein, which serves as a barrier against immune detection and destruction and helps cancer cells evade the immune system. EBV uses microRNAs to silence FOXP-1 and PBRM-1, lowering PD-L1 levels and can weaken the immune system’s ability to combat cancer by affecting NK cells’ combat capabilities. TLR (Toll-like receptor); FadA (*Fusobacterium* adhesion A); WNT (Wingless-related integration site); PD-L1 (Programmed death ligand 1); FOXP-1 (Forkhead Box protein 1); PBRM-1 (Polybromo 1). The figure was prepared using BioRender software. To generate the Figure, Biorender.com was used (accessed on 1 November 2024) [64,65,71,72,73].

Therefore, Mapping Cancer’s Path underscores the critical role of microbiota in shaping carcinogenesis. From gastrointestinal to systemic malignancies, understanding these microbial pathways enhances our knowledge of cancer biology and opens new avenues for diagnosis and therapy. This integrative approach highlights the importance of targeting microbial drivers to mitigate cancer risk and improve therapeutic outcomes (Figure 2). 

By utilizing genetic and environmental data to develop an all-encompassing cancer risk prediction model, Mapping Cancer’s Path is a noteworthy breakthrough in our understanding of cancer risk. Large-scale genomic sequencing, epigenetic modifications, and proteomic profiles are used in this method to identify mutations and biomarkers linked to different types of cancer. Researchers may create more accurate and individualized risk profiles by combining this genomic data with patient demographics, lifestyle variables, and environmental exposures [34]. Early identification and prevention are two of the main areas in which Mapping Cancer’s Path finds application. Healthcare practitioners can identify individuals who are more susceptible to certain cancers and develop screening programs, lifestyle changes, and preventative measures specifically for them. For example, those who have a hereditary susceptibility to breast cancer could benefit from routine mammograms or preventive surgery. Targeted therapy development is another benefit of Mapping Cancer’s Path. Through an awareness of the distinct molecular landscape of a patient’s tumor, medical professionals are able to select treatments that precisely target the pathways responsible for tumor development and resistance. This individualized strategy reduces unnecessary side effects while increasing therapeutic efficacy. Moreover, by focusing resources on high-risk groups and raising awareness of cancer preventive techniques, public health programs and policies can benefit from the insights gathered by Mapping Cancer’s Path. All things considered, this all-encompassing approach not only improves the treatment that patients receive on an individual basis but also supports larger initiatives in public health and cancer research, which eventually aim to lower the incidence and mortality of cancer [74].

### 5.3. Multi-Omics

Multi-omics is an extensive approach that combines multiple omics technologies, such as genomics, transcriptomics, proteomics, and metabolomics, to gain a comprehensive understanding of biological systems. The utilization of multi-omics has become extremely important in gut microbiota research as it provides a complete understanding of the complicated connections between microbial populations and the host. These strategies combine several forms of omics data. Metagenomics uncovers genetic potential in microbial communities. Metatranscriptomics is a technique used to identify the precise genes that are actively being transcribed in various conditions [75]. Metaproteomics is the scientific procedure of identifying and measuring proteins, which reveals valuable insights into their functional properties. Metabolomics is an area that focuses exclusively on metabolites, which are the end products of biological processes. By studying metabolites, we may obtain insights into the metabolic activities of the microbiota [76,77]. Collectively, these omics provide a thorough perspective on the functions of microbial organisms and their impact on the host’s health [78]. 

The gut microbiota, a diverse group of bacteria, significantly impacts metabolic processes in health and disease. Research using metagenomics and metatranscriptomics tools reveals its diverse functions and ecological specializations. The study suggests that the importance of gut microbiota may be underestimated due to their roles primarily within the gut. This approach is particularly useful for identifying metabolically active microorganisms in both healthy and pathological conditions, as their actions change significantly during transitions [79]. Metagenomics and proteomics are crucial tools for understanding gut microbiota at various molecular levels. They are integrated into studies to detect microbial populations and proteins in healthy and diseased states. For example, Crohn’s disease-related imbalance in gut bacteria triggers immune system responses, decreases bacteria-producing butyrate, and complicates the interaction between gut bacteria imbalances and type 2 diabetes. Additionally, metagenomics and metabolomics have been used to study the relationship between hosts and gut microorganisms [80]. Metagenomics and metabolomics are techniques used to understand gut microorganisms’ impact on health and disease. They identify microbial indicators or metabolites, aiding in early disease detection. Consuming a cafeteria diet can lead to obesity due to decreased bacteria and accumulation of hippuric acid, a biomarker linked to metabolic disorders. Personalized diets are crucial for managing gut flora. However, understanding complex gut substances and identifying specific species responsible for microbial metabolites remain a challenge [81,82].

These methods have the ability to locate biomarkers for diseases, which is crucial for understanding the mechanisms behind microbial dysbiosis in conditions like IBD and colorectal cancer. Proteomics has the capacity to detect variations in microbial protein expression related to diseases, while metabolomics can reveal changes in metabolic processes [51]. By incorporating distinct microbial activities and metabolic profiles into specific health issues, the integration of omics data has the potential to facilitate personalized treatment approaches, enabling customized therapies. Through the integration of data from different omics layers, researchers have the potential to establish a more accurate gut microbiome. This could lead to enhanced predictions regarding the impact of changes in the microbiota on both health and illness [83]. When conducting multi-omics network analysis in microbiome investigations, it is important to take into consideration the following methodological factors: Data integration is the necessary process of combining data from various omics layers, including genomics, transcriptomics, proteomics, and metabolomics. Successfully handling the complex structures and variations in the data necessitates the use of powerful statistical and computational techniques [84]. Ensuring the comparability of data from various omics platforms is of utmost importance, and normalization and standardization play a critical role in accomplishing this. Through the application of appropriate methods, biases can be minimized, and the quality of data can be improved.

### 5.4. Cutting-Edge Technology

Scientists initially used microscopes for microscopic observation to explore the vast world of microbes in our bodies. Later, they developed laboratory methods to grow and isolate individual microbes, allowing for a more detailed study of their diversity. In the current era, with the successive development of microbiome identification methods, e.g., (1) electrophoresis-based methods, such as denaturing gradient gel electrophoresis (DGGE), temperature gradient gel electrophoresis (TGGE), (2) PCR-based methods, restriction fragment length polymorphism (T_RFLP), random amplified polymorphic DNA (RAPD), fluorescence in situ hybridization (FISH), clone libraries, and gene chips [85], and (3) the emergence of high-throughput sequencing technologies, including amplicon sequencing, meta-omics, and shotgun sequencing, has expanded the understanding of microbial diversity and evolutionary relationships of microbiota [85,86].

Cutting-edge technologies have revolutionized cancer risk prediction by providing more precise and customized evaluations, especially AI and ML. Conventional techniques depend on broad risk factors such as genetic mutations, lifestyle choices, and family history; however, AI-driven models are able to combine large datasets from lifestyle, proteomic, and genomic data to reveal intricate, non-linear correlations between these variables. For example, AI technology may evaluate genetic sequencing data, identify novel biomarkers, and correlate them with environmental and behavioral factors to provide a deeper understanding of cancer risk [87]. A branch of artificial intelligence known as deep learning algorithms is especially adept at interpreting medical images, such as mammograms or computer tomography (CT) scans, to find early indications of cancer that human radiologists would overlook. Furthermore, AI can be used to forecast a patient’s specific biological profile-based response to therapy for a particular malignancy, resulting in more individualized preventative measures. These technologies have implications for public health beyond only diagnosis as they make it possible to identify people at high risk, which in turn enables focused screening and preventative measures. Therefore, proactive prevention rather than reactive therapy can become the primary emphasis of healthcare with AI-powered cancer risk prediction. Large-scale data analysis and predictive modeling together offer a paradigm shift that makes tailored risk assessments, earlier detection, and higher survival rates possible. For broad adoption, there are still issues with data privacy, model transparency, and incorporating AI into clinical procedures that need to be resolved [88].

## 6. Integrating Technologies to Analyze Gut Microbiota for Cancer Risk Assessment

Research indicates that the microbiome’s composition may influence diseases such as cancer, necessitating further study on the human gut microbiome. Dysbiosis in gut microbiota has been linked to the start and progression of CRC [89]. New technology in analyzing gut microbiota can improve disease diagnosis without invasive procedures. High-throughput approaches, such as NGS, enhance the validity of cancer and microbiome examinations. Different methods can examine gut microbiota attributes to identify correlations with cancer signs. The 16S rRNA gene sequencing approach is effective in evaluating bacteria diversity and identifying alterations in the microbiota composition related to cancer. This approach enhances the validity of cancer diagnosis and microbiome examinations. Whole Metagenome Sequencing (WMS) is a technique that analyzes all of the genetic material in a sample, enabling a thorough comprehension of the microbiota’s biological functions. This approach enables the discovery of genes related to metabolic pathways, antibiotic resistance, and virulence factors, which could impact the development of cancer and the efficacy of treatment [90]. Metatranscriptomics and metaproteomics are two scientific techniques used to analyze the metabolic pathways and activities of the microbiota. Metatranscriptomics identifies highly expressed microbial genes over specific periods and situations, while metaproteomics detects and analyzes proteins produced by the microbiome, affecting biological processes such as inflammation, immunological regulation, and drug metabolism. Metabolomics analyzes small molecules and metabolites released by the microbiota, identifying substances that can modify host metabolic pathways [91].

Several types of bacteria, such as *F. nucleatum*, *Bacteroides fragilis*, *E. coli*, *Peptostreptococcus*, *Porphyromonas*, *Bacteroides*, and *Streptococcus*, were identified as contributing to inflammation and the development of CRC. These bacteria are associated with multiple processes, such as LPS and energy production, protein and mucin catabolism, and carbohydrate degradation, providing important information about their function in CRC. *F. nucleatum* can adhere to and infiltrate colon epithelial cells, triggering inflammation and tumor development. *E. coli* and *Bacteroides fragilis* are linked to DNA damage and have been found to promote tumorigenesis by activating inappropriate immune responses by Th17 cells in animal models of CRC [92].

Studies have shown that gut microbiota can affect the liver by activating the immune system and causing deterioration of liver cells through necrosis and apoptosis, leading to significant fibrosis and potentially resulting in liver cancer. The gut microbiota can be used as a non-invasive biomarker for early detection of hepatocellular carcinoma (HCC). Researchers have used NGS for examining fecal samples to identify microbial species associated with HCC occurrence and progression [93]. Metagenomic sequencing provides a complete understanding of the roles and metabolic processes of microbiota. Some bacteria, such as *E. coli* and *Enterococcus faecalis*, produce toxins that may cause liver damage and the development of HCC. *Bacteroides* and *Clostridium* can exacerbate inflammation and contribute to cancer progression by inducing inflammation in the liver. Changes in the gut microbiome makeup, with lower levels of *Firmicutes* and a rise in *Proteobacteria*, exist in patients with HCC [94]. The gut microbiota produces important compounds that have significant effects on HCC development as biomarkers. SCFAs, including butyrate, produced by gut microorganisms, have anticancer properties by stimulating programmed cell death and lowering inflammation. Gut microbiota may change bile acids, which may lead to liver cancer development. Indole derivatives, formed by microbial metabolism of tryptophan, have anti-tumor effects and can act as markers for imbalances in gut microbiota and HCC [95,96].

**Figure 3:**
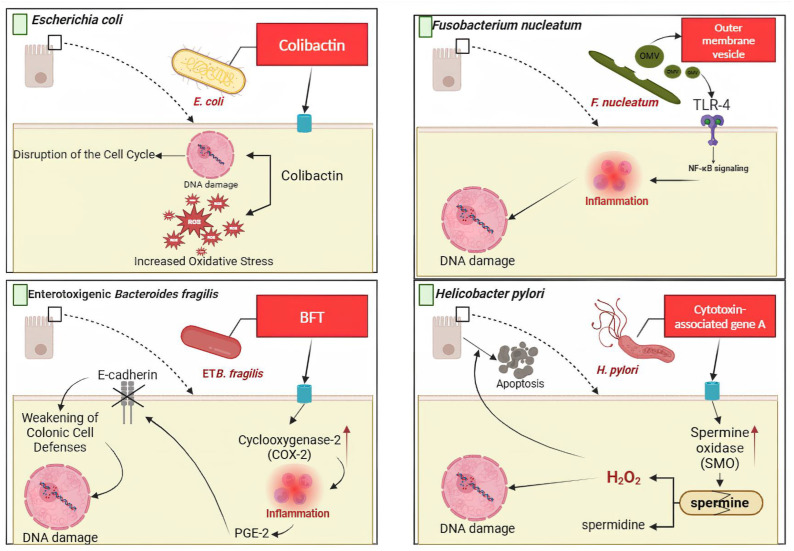
The intricate pathways of *E. coli*, *F. nucleatum*, *B. fragilis*, and *H. pylori* contribute to DNA damage and colon inflammation. *E. coli*, *B. fragilis*, and *F. nucleatum* are important players in inflammation and the development of CRC. These bacteria contribute to the pathophysiology of CRC by producing LPS, protein catabolism, and breaking down carbohydrates. *F. nucleatum* in CRC triggers inflammation and carcinogenesis, while *B. fragilis* and *E. coli* increase tumor development and DNA damage through Th17 cell-mediated immune responses. *H. pylori* causes stomach cancer by breaking down the stomach lining, producing toxic H_2_O_2_, leading to DNA damage and programmed cell death. Moreover, *H. pylori* results in increased levels of PD-L1 expression in stomach lining cells, enabling them to evade immune recognition, potentially contributing to cancerous growth. ETB (Enterotoxigenic *Bacteroides*); BFT (*Bacteroides fragilis* toxin); PGE-2 (Prostaglandin E2); OMV (Outer membrane vesicles); TLR-4 (Toll-like receptor 4); NF-kB (Nuclear factor kappa B). The figure was made using BioRender software. To generate the Figure, Biorender.com was used (accessed on 1 November 2024) [92,94].

The gut microbiota plays a significant role in the development and progression of ovarian cancer, as it promotes intestinal inflammation and activates tumor-related signaling pathways [97]. A study using 16S rRNA Gene sequencing and metagenomics predicted the functional potential of gut microbiota, including its role in lactate generation and use. The research suggests that gut microbiota can serve as a biomarker to evaluate the success of ovarian cancer treatments. Patients who respond well to chemotherapy have a more diverse and stable gut microbiota, while those resistant have a more limited microbiota. The presence of lactate-producing bacteria in patients sensitive to platinum-based treatments may enhance the effectiveness of medical treatments [98]. A diverse and stable gut microbiota could improve treatment outcomes. Restoring the proper balance of microorganisms in the gut through probiotics, prebiotics, or fecal microbiota transplantation could lower the risk of cancer and improve the success rate of cancer treatments [99]. (Figure 3).

## 7. Conclusions

The interplay between human health and gut microbiota is crucial for understanding cancer, particularly colorectal cancer, influenced by dysbiosis (Table 2). Advanced technologies, such as next-generation sequencing and multi-omics, have enabled precise microbiome profiling, improving early detection and risk assessment through microbial signatures. Artificial intelligence further enhances the potential of microbiota manipulation as a therapeutic tool, while microbial biomarkers offer promising avenues for targeted therapies and non-invasive diagnostics. As these technologies evolve, they will clarify the gut microbiota’s role in cancer, paving the way for innovative treatments and improved patient outcomes, underscoring the need for a multidisciplinary approach in oncology.

## Figures and Tables

**Table 1:** Advanced technologies for analyzing gut microbiota and predicting cancer risk.

Technology [Ref.]	16S rRNA Sequencing [18,19]	Metagenomics [20,21]	Metatranscriptomics [22]	Metaproteomics [23,24]	Metabolomics [25,26]
Approach	Sequencing of 16S rRNA genes	Whole-genome sequencing	Sequencing of RNA transcripts	Proteomic analysis	Metabolite profiling
Sample Requirements	DNA	High-quality DNA	High-quality RNA	Protein extraction	Stable metabolite extraction
Analysis of Gut Microbiota	Bacterial composition	Microbiota profiling	Microbial functions	Protein expression	Microbiota metabolites
Role in Predicting Cancer	Correlation of bacteria with cancer	Detects genetic elements in cancer	Links microbial gene expression and cancer	Identifies biomarkers for cancer	Analyzes metabolic pathways in cancer
Time to Results	Days to weeks	Weeks	Weeks	Weeks	Days to weeks
Limitations	Limited to bacterial identification	Expensive; complex data interpretation	RNA degradation requires hard sample handling	Requires specialized equipment; protein complexity	Variability in metabolite concentrations; data interpretation
Sensitivity/Specificity	Moderate to high	High	High	High	Moderate
Standardization/Reproducibility	High	Variable	Moderate to high	Low to moderate	Variable
Applications	Cancer risk based on microbial profiles	Gut microbiomes’ role in cancer	Microbiota metabolic activity in cancer	Biomarker discovery for cancer detection	Microbial metabolites in cancer prognosis

**Table 2:** Summary of Sequencing Methods and Technologies: Strengths, Weaknesses, Limitations, and Critical Analysis.

Aspect	Strengths	Weaknesses	Limitations	Possible Problems	Critical Analysis
Sequencing Platforms					
NGS	High throughput and accuracy, Cost-effective, Versatile applications	Complex data analysis High computational need	Short read lengths, limited resolution for structural variants, library preparation complex	Sample contamination data analysis challenges sequencing artifacts	Providing high throughput and accuracy
Bioinformatic Data Analysis Methods					
Meta-omics	Comprehensive profiling Functional insights	Data complexity, bioinformatics support	Complex data interpretation High costs, rare taxa limitation	Function overlap issues sample contamination risks	Requires complex analysis and bioinformatics expertise
OTU	Simplifies classification, Facilitates comparative studies, reduces noise in data	Limited resolution, dependence on reference databases	Intra-genomic variability, no quantitative insights, Environmental contamination	Dependence on accurate classification, rare taxa overlook	Simplifies classification but is limited in resolution and quantitative insights
**ASV**	High-resolution analysis Rare variant detection PCR-free options	Requires sophisticated analysis, PCR biases	Reference quality dependence, variant limitations	Computational analysis demands limited sensitivity for low-frequency variants	High-resolution option but needs computational resources
Machine Learning Techniques					
SVM	Effective in classification and regression, handles high-dimensional data	Requires careful parameter tuning, may struggle with very large datasets without optimization	Computationally expensive for large datasets, sensitive to noisy data	Overfitting, data preprocessing challenges, sensitivity to noisy or unbalanced data	Powerful in classification tasks but requires optimization and computationally expensive for large datasets.
SHAP	Enhances interpretability of complex models, identifies feature importance	High computational cost depends on model complexity and feature interactions	Limited to interpretation without improving model accuracy	Interpretation may vary across models, computational demands for large data sets	SHAP improves model transparency, offering insight into feature influence, but requires significant computation.
Emerging Technologies					
Multi-omics	Integrative analysis across omics layers, comprehensive systems biology insights	Requires complex data integration, high bioinformatics expertise, large-scale data	High costs, complexity in managing heterogeneous data	Data harmonization challenges, computational demands	Multi-omics provides in-depth biological insights but requires substantial data integration
Cutting-Edge Technology	Potential for real-time results, Enhanced resolution, Interdisciplinary uses	May lack validation, high costs, Scalability issues	Implementation challenges Limited reproducibility	Rapid advancements data management issues infrastructure demands	Offer improved resolution but face issues in validation and cost.
Amplicon Sequencing	Targeted sequencing, High specificity, Cost-effective,	PCR bias, short read lengths, limited for comprehensive studies	Limited structural variants Coverage bias, PCR artifacts	PCR amplification bias low rare variant representation	Useful for targeted insights but limited by PCR biases
16S rRNA Gene Sequencing	Broad taxonomic coverage Cost-effective, Established protocols	Prokaryotes limited, short read lengths, No functional insights, Primer bias	Limited taxonomic resolution, no functional insights	Low closely related taxa resolution, high PCR bias risks	Useful for microbial profiling but lacks resolution and functional information.
Mapping Cancer’s Path	Provides insights into tumor evolution, Personalized medicine, Comprehensive profiling	High complexity, requires specialized skills, tumor heterogeneity	Potentially incomplete data high costs, regulatory complexity	Complexity of genomic data, interpretation challenges	Data complexity and interpretation pose challenges

## Data Availability

Not applicable.

## Abbreviations

AI	Artificial Intelligence
ASV	Amplicon Sequence Variant
CRC	Colorectal Cancer
DCA	Deoxycholic Acid
DGGE	Denaturing Gradient Gel Electrophoresis
FAP	Familial Adenomatous Polyposis
FISH	Fluorescence In situ Hybridization
FMT	Fecal Microbiota Transplantation
GEO	Gene Expression Omnibus
HCC	Hepatocellular Carcinoma
IBD	Inflammatory Bowel Disease
ITS	Internal Transcribed Spacer
LTA	Lipoteichoic Acid
ML	Machine Learning
NGS	Next-Generation Sequencing
OUT	Operating Taxonomic Unit
RAPD	Random Amplified Polymorphic DNA
SCFAs	Short-Chain Fatty Acids
SVM	Support Vector Machine
TCMA	The Cancer Microbiome Atlas
TGGE	Temperature Gradient Gel Electrophoresis
TMAO	Trimethylamine N-Oxide
T-RFLP	Terminal-Restriction Fragment Length Polymorphism
WGSS	Whole-Genome Shotgun Sequencing
WMS	Whole Metagenome Sequencing
XGBoost	eXtreme Gradient Boosting
